# The Association Between Older Adult Technology Use and Mental Health During the COVID-19 Pandemic

**DOI:** 10.1093/geroni/igab046.2163

**Published:** 2021-12-17

**Authors:** Brittany Drazich, Nancy Perrin, Laura Samuel, Melissa diCardi Hladek, Sarah Szanton, Thomas Cudjoe, Janiece Taylor, Qiwei Li

**Affiliations:** 1 Johns Hopkins University School of Nursing, Baltimore, Maryland, United States; 2 Johns Hopkins University, Baltimore, Maryland, United States; 3 Johns Hopkins University School of Medicine, Baltimore, Maryland, United States; 4 Johns Hopkins School of Nursing, Baltimore, Maryland, United States

## Abstract

Physical distancing during the COVID-19 pandemic may impact the mental health of older adults, but technology use may buffer this impact. This study aimed to 1) examine changes in older adult technology use during the COVID-19 pandemic and 2) determine if technology use moderates the relationships between decreased in-person communication/activity and the mental health of older adults during the pandemic. Data were taken from the NHATS COVID-19 Round 10 (n= 3,188). Older adults engaged in more technology-based activity (b= .237, p<0.001), technology-based healthcare communication (b=.112, p<0.001), and technology-based food acquisition (b= .214, p<0.001) during the COVID-19 pandemic, compared to before. Technology use did not moderate the relationship between decreased in person personal communication (b= .021, p= 0.662)/activity (b= .045, p= 0.749) and mental health during the pandemic. Although older adults are utilizing technology more during the pandemic, it might not be protective against negative mental health outcomes from physical distancing.

